# Enhanced target cell specificity and uptake of lipid nanoparticles using RNA aptamers and peptides

**DOI:** 10.3762/bjoc.17.75

**Published:** 2021-04-26

**Authors:** Roslyn M Ray, Anders Højgaard Hansen, Maria Taskova, Bernhard Jandl, Jonas Hansen, Citra Soemardy, Kevin V Morris, Kira Astakhova

**Affiliations:** 1 Center for Gene Therapy, Beckman Research Institute, City of Hope, Duarte, CA, United States of America; 2 Department of Chemistry, Technical University of Denmark, Lyngby, Denmark; 3 Institute of Biological Chemistry, Faculty of Chemistry, University of Vienna, 1090 Vienna, Austria; 4 School of Medical Sciences, Griffith University, Gold Coast, Australia 4222; 5 Menzies Health Institute Queensland, Griffith University, Gold Coast, QLD 4222, Australia

**Keywords:** aptamer, blood–brain barrier, gene therapy, HIV-1, lipid nanoparticle

## Abstract

Lipid nanoparticles (LNPs) constitute a facile and scalable approach for delivery of payloads to human cells. LNPs are relatively immunologically inert and can be produced in a cost effective and scalable manner. However, targeting and delivery of LNPs across the blood–brain barrier (BBB) has proven challenging. In an effort to target LNPs composed of an ionizable cationic lipid (DLin-MC3-DMA), cholesterol, the phospholipid 1,2-distearoyl-*sn*-glycero-3-phosphocholine (DSPC), and 1,2-dimyristoyl-*rac*-glycero-3-methoxypolyethylene glycol-2000 (DMG-PEG 2000) to particular cell types, as well as to generate LNPs that can cross the BBB, we developed and assessed two approaches. The first was centered on the BBB-penetrating trans-activator of transcription (Tat) peptide or the peptide T7, and the other on RNA aptamers targeted to glycoprotein gp160 from human immunodeficiency virus (HIV) or C-C chemokine receptor type 5 (CCR5), a HIV-1 coreceptor. We report herein a CCR5-selective RNA aptamer that acts to facilitate entry through a simplified BBB model and that drives the uptake of LNPs into CCR5-expressing cells, while the gp160 aptamer did not. We further observed that the addition of cell-penetrating peptides, Tat and T7, did not increase BBB penetration above the aptamer-loaded LNPs alone. Moreover, we found that these targeted LNPs exhibit low immunogenic and low toxic profiles and that targeted LNPs can traverse the BBB to potentially deliver drugs into the target tissue. This approach highlights the usefulness of aptamer-loaded LNPs to increase target cell specificity and potentially deliverability of central-nervous-system-active RNAi therapeutics across the BBB.

## Introduction

Lipid nanoparticles (LNPs) represent an effective platform for delivering small molecules, RNA, or DNA into target cells [1]. LNPs have been successfully deployed via different administration routes in vivo to distribute cargo into target tissues [2–8]. By changing lipid composition [6] and/or including short peptides [9] and ligands [10], one can modulate the biodistribution of the LNP in the body. However, despite these advances, targeting of LNPs to the brain tissue remains a challenge [11].

**Figure 1:**
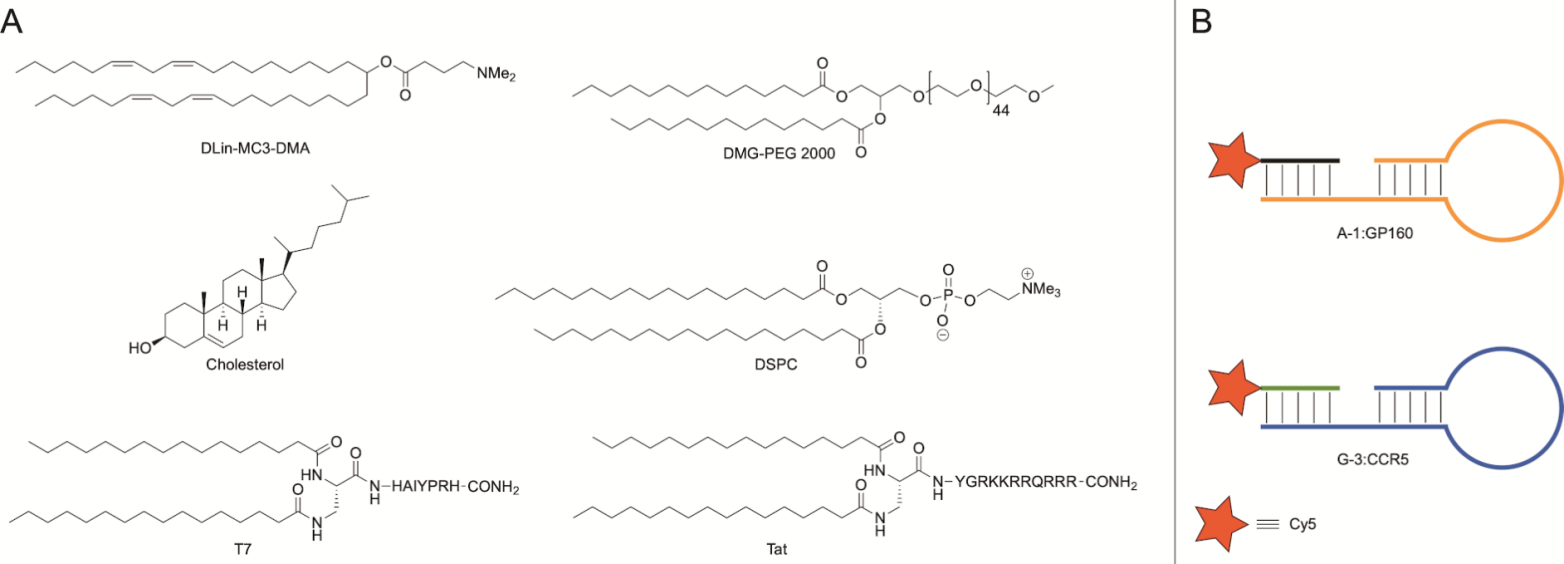
Components of the LNPs. A) Lipid species and lipidated cell-penetrating peptides applied by postinsertion. B) Graphical representation of aptamer–probe hybrids.

In order to reach safer therapeutic options for treatment of brain diseases and disorders, a productive drug transport across the blood–brain barrier (BBB) is critical. For example, despite successful implementation of antiretroviral drugs for the treatment of human immunodeficiency virus 1 (HIV-1), HIV-1-associated neurological disorders persist due to the poor uptake of antiretroviral drugs across the BBB [12–14]. There are two ways to traverse the BBB, one is through temporary disruption of the physical barrier, which impairs BBB function, and the other is to use nanocarriers or particles [11]. The latter presents a noninvasive route that is safer than physical disruption [11]. One approach to increase transport of LNPs through the notoriously protective BBB is to use short positively charged peptides or receptor-specific ligands, both of which have shown to be effective at increasing transport of LNPs, nucleotides, and small molecules through the BBB [9,15–17]. For example, the short positively charged peptide Tat has previously been demonstrated to be effective as an excipient species to increase the uptake through the negatively charged BBB [9,18]. Tat (sequence: H-YGRKKRRQRRR-NH_2_) is an arginine-rich short cell-penetrating peptide derived from the natural nuclear Tat protein of HIV-1 [19–20]. The HIV-1 Tat protein itself has been shown to traverse the BBB by acting as a cell-penetrating peptide [9,20]. Other small positively charged molecules used for BBB penetration include transferrin and corresponding peptide derivatives or analogs that act as ligands for the transferrin receptor. The transferrin receptor is highly expressed in brain capillaries, nucleated cells, and in rapidly dividing cells [21], and its endogenous ligand transferrin has previously been used to increase transport of small molecules and oligonucleotides across the BBB [21–23]. The peptide T7 consisting of seven amino acids (H-HAIYPRH-NH_2_) was identified via phage display [24] and has a high affinity (≈10 nM) for the transferrin receptor [24–25]. This peptide does not compete with endogenous transferrin binding and has been used to successfully enhance drug delivery to brain tissue [15,22,24–26]. Both peptides were included in this study and modified with an N-terminal lipid anchor for LNP postinsertion. The design of the lipid anchor includes two palmitoyl chains that are attached through a 1,2-diaminopropanoic acid moiety (Dap) on the N-terminus of each peptide, providing the lipidated peptides dipalmitoyl-Dap-T7 and dipalmitoyl-Dap-Tat (Figure 1A). Double lipidation ensures a more stable lipid-membrane-anchoring compared to a single fatty acid chain or cholesteryl variant [27–29]. The careful choice of Dap and palmitic acid allows for the entire synthesis to be performed on solid support with no need for additional reactions after cleavage [27–29].

One approach to generate LNP formulations with higher specificity for antigen-expressing cells is to use RNA aptamers. RNA aptamers are short oligonucleotides that are evolved using a process called systematic evolution of ligands by exponential enrichment (SELEX) [30]. SELEX is an iterative process that begins with a large oligonucleotide library that, through a process of negative and positive selections, ends with a few candidates that are specific for a particular protein [30–31]. Using HIV-1 as our model, we explored the use of two RNA aptamers as a mean to increase the specificity of LNPs for HIV-1-infected and/or target cells [31]. RNA aptamers are ideal candidates due to the lower immunogenicity profile than the DNA counterparts [30,32–33]. RNA aptamers are also highly amenable to form complex and dynamic secondary structures, which makes them ideal molecules for novel ligand development [31]. Zhou et al. previously reported on an RNA aptamer specific for the HIV-1 entry coreceptor C-C chemokine receptor type 5 (CCR5) [34] and an RNA aptamer specific for the HIV-1 envelope protein gp160 [35]. The CCR5 RNA aptamer G-3 has been shown to be specific for, and internalized by the CCR5 receptor [34]. Similarly, it has been found that the A-1 aptamer specifically recognizes gp160 and that it may be internalized through receptor-mediated endocytosis [35]. Both the G-3 and A-1 aptamers have been conjugated to small interfering RNA (siRNA), through a stick bridge motif, to deliver siRNAs into the respective target cells. The G-3 siRNA conjugate had the highest efficacy with 70% delivery into target T-cells, while the A-1 siRNA conjugate showed a 20% delivery into target gp160-expressing cells [34–35]. Thus, both aptamers present an additional potential route for LNP internalization and target cell specificity. In order to assess the ability of aptamers to drive LNP internalization, short complementary Cy5-DNA oligonucleotides specific for each aptamer were used as probes to detect LNP uptake in different cells.

In this study, we employed lipid compositions and formulation procedures previously reported in literature [4]. Specifically, the cationic and ionizable DLin-MC3-DMA lipid is a constituent of the FDA-approved LNP-formulated siRNA drug Patisiran^®^ for treatment of familial transthyretin amyloidosis [36–37]. Clinical trial safety assessments of this formulation showed no liver toxicity and no immune stimulation, with ≈10% of trial participants experiencing mild to moderate adverse events upon administration [38]. It includes encapsulation of siRNA by a mixture of lipid components, such as an ionizable cationic lipid, 1,2-distearoyl-*sn*-glycero-3-phosphocholine (DSPC), cholesterol, and PEG-lipid, each with an essential role in the design (Figure 1). These lipids promote the effective distribution of the LNP in vivo as well as aid in effective cargo release from the endosome [1,37]. To this end, we herein report the efficacy, delivery capability, and functionality of the addition of peptides and RNA aptamers in facilitating entry through a simplified BBB model as well as to determine whether inclusion of these molecules could facilitate cell specific uptake. We further show that LNPs generally exhibit a low immunogenic and toxic profile and that RNA aptamers can act as potential enhancers to effectuate the delivery of LNPs into the central nervous system (CNS).

## Results

### Lipid nanoparticle development and characteristics

In accordance with a previously published procedure, we generated LNPs using a mixture of DLin-MC3-DMA, DSPC, cholesterol, and 1,2-dimyristoyl-*rac*-glycero-3-methoxypolyethylene glycol-2000 (DMG-PEG 2000). Lipids were first extruded and then complexed with negatively charged aptamers annealed with fluorescently tagged complementary DNA oligonucleotides (GP160:A-1 or CCR5:G-3) to simultaneously assemble the LNPs (the formulation list is provided in Table 1). At this stage, the LNPs were examined by dynamic light scattering (DLS, Table 2). While noncomplexed (empty) LNPs had an average size of 62.4 nm and a zeta potential (ZP) of −2.9 mV, LNPs mixed with GP160:A-1 and CCR5:G-3 displayed average sizes of 57.3 nm and 91.9 nm, respectively, as well as a more negative ZP (−11 mV and −9.4 mV, respectively, Table 2). These ZP values indicate that complexation leads from a neutral to anionic LNP product [39], a property that typically confers with low to no cytotoxicity in vivo [40]. Further, the additional decrease in the ZP indicates efficient aptamer loading into the LNPs. Additionally, low polydispersity index (PDI) values reported for both formulations (Table 2) indicate a high degree of monodispersity.

**Table 1:** Formulations used in the present study.^a^

LNP sample	Cy5 DNA probe/aptamer	lipopeptide

LNP B9	—	—
LNP B9 A-1	A-1:GP160	—
LNP B9 G-3	G-3:CCR5	—
LNP B9 T7	—	T7
LNP B9 Tat	—	Tat
LNP B9 A-1 T7	A-1:GP160	T7
LNP B9 A-1 Tat	A-1:GP160	Tat
LNP B9 G-3 T7	G-3:CCR5	T7
LNP B9 G-3 Tat	G-3:CCR5	Tat

^a^T7 (H-HAIYPRH-NH_2_) is targeting transferrin receptor. Tat (H-YGRKKRRQRRR-NH_2_) is derived from the natural nuclear Tat protein of HIV-1.

**Table 2:** DLS data listing particle size, PDI, and ZP of LNP formulations.

LNP formulation	physical characterization by DLS		

	mean diameter (nm)	PDI	zeta potential (mV)

LNP B9	62.4 ± 0.7	0.2 ± 0.01	−2.9 ± 1.1
LNP B9 A-1	57.3 ± 0.9	0.1 ± 0.03	−11.0 ± 1.4
LNP B9 G-3	91.9 ± 4.2	0.3 ± 0.03	−9.4 ± 1.0

Next, LNPs were incubated with either Tat or T7 and the physical characteristics assessed by nanoparticle tracking analysis (NTA, Table 3) and transmission electron microscopy (TEM, Supporting Information File 1, Figure S2). After postinsertion, LNP sizes were found by NTA to range from 54–66 nm (Table 3), while TEM analysis revealed average sizes between 45–52 nm (Supporting Information File 1, Figure S2B). While there appears to be a ≈10 nm discrepancy when comparing DLS and NTA with TEM, this size difference was found to be consistent between these methods of analyses for all samples.

**Table 3:** NTA analysis listing size and concentration of LNPs.

LNP formulation	physical characterization by DLS		

	mean diameter (nm)	standard deviation (nm)	particle concentration ± SEM (particles/mL)

LNP B9	69.2 ± 0.3 nm	30.8 ± 1.5 nm	3.13 × 10^11^ ± 1.75 × 10^10^
LNP B9 A-1	66.6 ± 1.4 nm	25.2 ± 1.4 nm	3.82 × 10^11^ ± 6.20 × 10^9^
LNP B9 A-1 T7	65.7 ± 1.1 nm	26.3 ± 2.4 nm	3.25 × 10^11^ ± 2.82 × 10^10^
LNP B9 A-1 Tat	54.2 ± 0.6 nm	22.1 ± 1.4 nm	8.90 × 10^11^ ± 7.23 × 10^10^
LNP B9 G-3	67.2 ± 0.3 nm	30.2 ± 0.8 nm	2.71 × 10^11^ ± 1.45 × 10^10^
LNP B9 G-3 T7	66.5 ± 1.7 nm	32.2 ± 5.0 nm	3.30 × 10^11^ ± 2.60 × 10^10^
LNP B9 G-3 Tat	57.3 ± 0.5 nm	29.2 ± 1.7 nm	8.05 × 10^11^ ± 7.83 × 10^10^
LNP B9 T7	75.1 ± 1.5 nm	32.0 ± 1.4 nm	2.19 × 10^11^ ± 1.65 × 10^10^
LNP B9 Tat	61.2 ± 0.7 nm	15.2 ± 1.5 nm	2.19 × 10^11^ ± 1.69 × 10^10^

For example, LNP B9 A-1 Tat was characterized by the smallest average size using both NTA (≈54 nm) and TEM (≈45 nm). Thus, the average sizes obtained by NTA are in agreement with the average size observed using TEM (Supporting Information File 1, Figure S2A and Table S2). Similarly, while the mean diameter of LNP B9 G-3 was found to be larger by DLS (91.9 nm) than the values reported for NTA (67.2 nm) and TEM (52 nm), the sizes of the LNP B9 and LNP B9 A-1 samples via DLS are also in agreement with the reported NTA and TEM sizes. These discrepancies may be indicative of the inherent differences between these three analytical methods and highlight the need to confirm LNP sizes using more than one technique. Nevertheless, the small size of these nanoparticles (<100 nm) is ideal for in vivo applications as they may bypass the reticuloendothelial system and thereby increase LNP circulation time in vivo [41].

### LNPs with postinsertion T7 peptide

Previous studies have demonstrated the ability of the T7 peptide to increase LNP transport across the BBB [22–24,42]. In order to test this, we used hCMEC/D3, HEK293Ts, HeLa, and TZM-bL cell lines. hCMEC/D3 is a human brain endothelial cell line that mimics a simplified BBB and, using a transwell assay, allows to study the BBB penetration potential of compounds [43]. To assess the specific uptake of the G-3 aptamer, we used TZM-bLs. TZM-bL is a HeLa-derived cell line that was engineered to express CD4 and CCR5 receptors on the cell surface [44]. HeLa cells were used as a negative control. To investigate the specific uptake of the A-1 aptamer, we used a HEK293T cell line engineered to express gp160 [45], HEK293T-gp160, and the parental HEK293T served as a control cell line.

**Figure 2:**
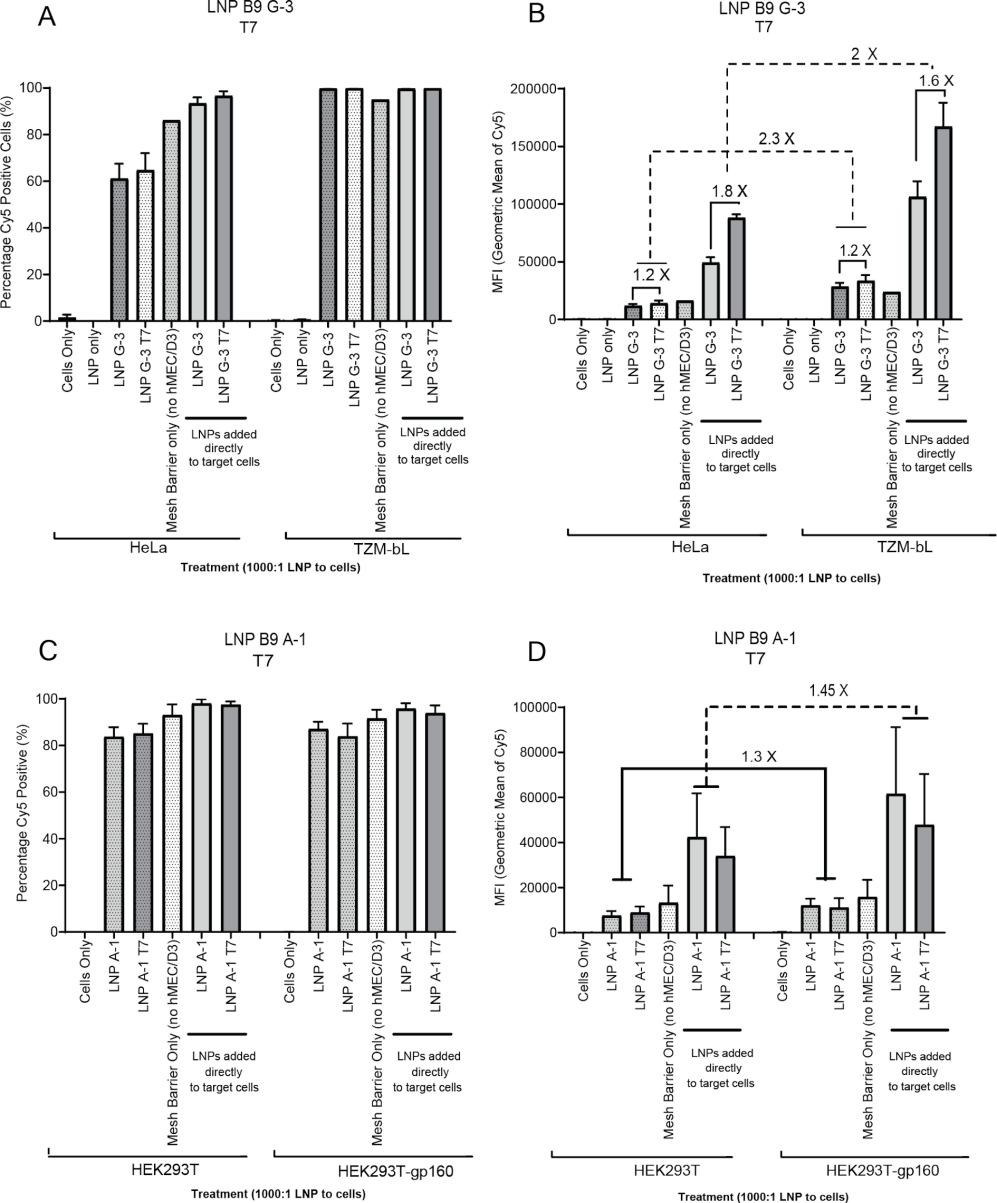
LNPs with T7 pass through the transwell cell barrier and are taken up by target cells. HeLa (CCR5-negative control cell) or TZM-bl (CCR5-positive cell type) cells (A and B) as well as HEK293T or gp160 positive HEK293T cells (C and D) were seeded at a density of 50,000 cells/well. The next day, transwell inserts containing confluent hMEC/D3 cells at trans-endothelial electrical resistance (TEER) above 30 Ω⋅cm^2^ were placed into experimental wells, LNPs (1000:1) were added to the apical surface, and 24 h later, the target cells were processed for Cy5 detection using fluorescence-activated single-cell sorting (FACS). A) Percentage of cells positive for Cy5 detection in HeLa and TZM-bls. B) MFI of Cy5 in each cell population in HeLa and TZM-bls. C) Percentage of cells positive for Cy5 detection in HEK cell types. D) MFI of Cy5 in each cell population in HEK cell types. Histograms are representative of three independent biological experiments, each containing duplicate technical replicates.

hCMEC/D3 cells were cultured on a 0.4 µM transwell mesh until a trans-endothelial electrical resistance of above 30 Ω⋅cm^2^ was reached. This measure is an indicator that a tight junction barrier has formed within these cells and can be used to determine the ability of the LNPs to pass through the BBB (Supporting Information File 1, Figure S3A). Additionally, we further confirmed our junctions using fluorescent microscopy on the barrier layers to confirm expression of claudin-5, a known tight junction protein (Supporting Information File 1, Figure S3B). We observed that LNPs were readily taken up by both HeLa and TZM-bls in the absence of a transwell insert (Figure 2A and Figure 2B). With the addition of the hCMEC/D3 cells in the apical chamber, we found that HeLa cells were less Cy5-positive (≈60%) than the target TZM-bl cells (≈100%, Figure 2A). Further, when examining the intensity of Cy5 in these cell populations, we found that the addition of the T7 peptide increases uptake by 1.2-fold through the hCMEC/D3 cellular barrier while also increasing uptake through direct addition by 1.6–1.8-fold (Figure 2B). Additionally, the mean fluorescent intensity (MFI) was found to be 2–2.3-fold higher in the target TZM-bl cells in both barrier and nonbarrier treatment groups compared to the control HeLa cells, indicating a higher accumulation of LNPs in the target cells (Figure 2B and Supporting Information File 1, Figure S4). Passive diffusion of the LNPs with the G-3 aptamer alone through a transwell insert without hCMEC/D3 cells appears to show higher uptake in the HeLa cell line but lower uptake in the TZM-bl cell line in comparison to the transwell insert with hCMEC/D3 cells (Figure 2A and Figure 2B).

In contrast, we found that formulating LNPs with the gp160-specific A-1 aptamer did not result in any significant increase in percentage uptake in the target gp160-positive HEK293T cells compared to HEK293T cells alone (Figure 2C). However, we did observe the MFI in gp160-positive HEK293T to be 1.3- and 1.45-fold higher (barrier and nonbarrier groups, respectively) than in the HEK293T cells alone (Figure 2D), suggesting higher levels of LNPs in gp160-expressing HEK293T cells. We also observed that direct addition of the LNPs resulted in a higher percentage of Cy5-positive cell detection and a higher MFI compared to the hCMEC/D3 barrier (Figure 2D and Supporting Information File 1, Figure S4).

Collectively, these data suggest that the candidate LNPs, particularly LNP B9 G-3 T7, may increase uptake through tight junctions and prove useful in transiting drugs and small cargo through the BBB in vivo.

### LNPs with postinserted Tat peptide

**Figure 3:**
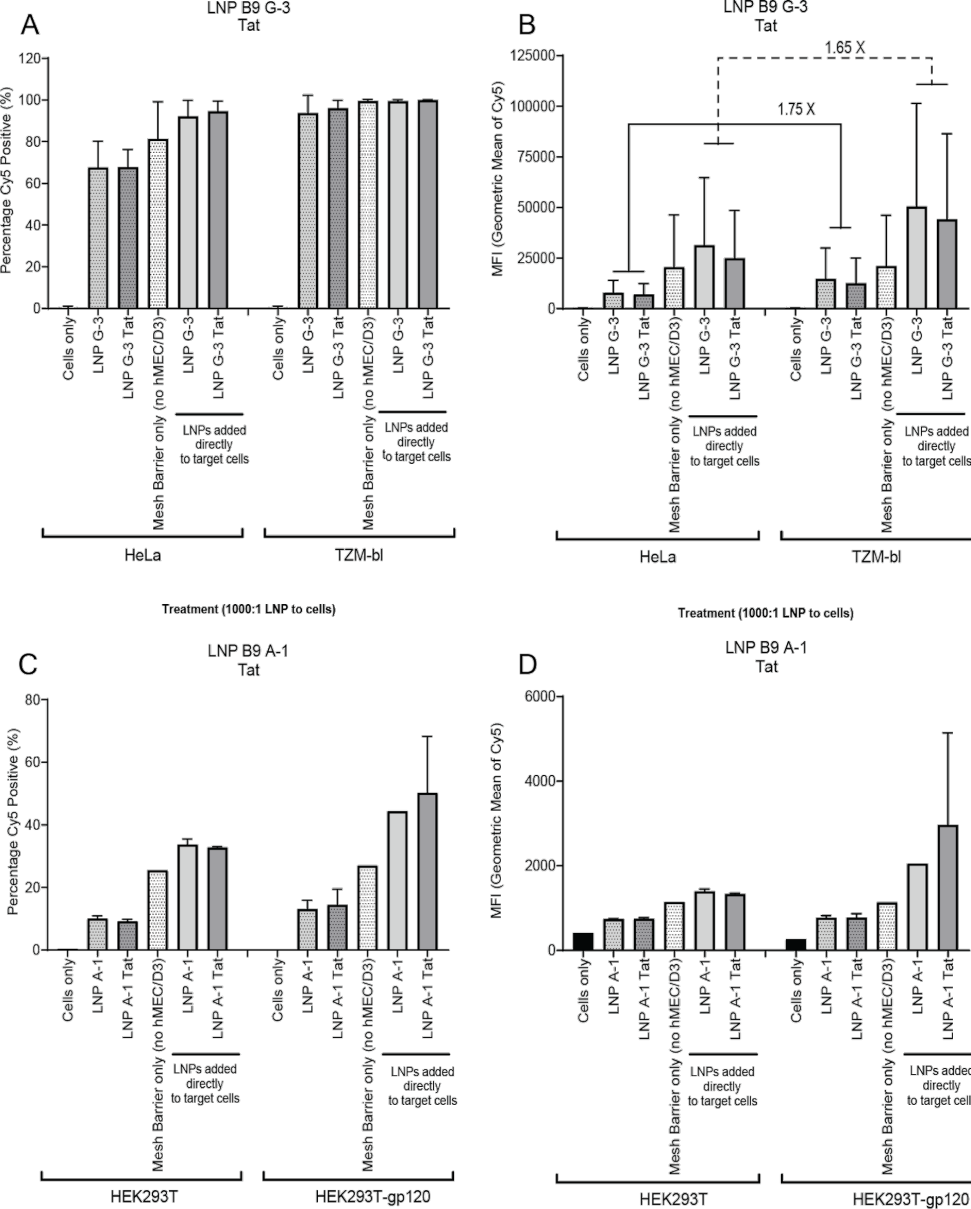
LNPs with Tat pass through the transwell cell barrier and are taken up by target cells. A) Percentage of cells positive for Cy5 detection in HeLa and TZM-bls. B) MFI of Cy5 in each cell population in HeLa and TZM-bls. C) Percentage cells positive for Cy5 detection in HEK cell types. D) MFI of Cy5 in each cell population in HEK cell types. Histograms are representative of two independent biological experiments, each containing duplicate technical replicates.

Tat is a cationic peptide that is known to increase transport of molecules through the BBB and increase uptake into cells [18]. In a similar manner to the transferrin peptide T7, we investigated the ability of Tat to drive LNP uptake in cell lines. Interestingly, we found that the addition of the Tat peptide to either the A-1- or G-3-complexed LNPs did not have any effect on BBB penetration (Figure 3). Rather, we observed that LNPs containing the G-3 aptamer showed an increased uptake in target cells expressing CCR5 (Figure 3A and Figure 3B). We observed that TZM-bls had a ≈98% uptake of LNPs via the hCMEC/D3 barrier compared to ≈63% in HeLa cells (Figure 3A). We also observed a similar increase (1.75-fold, barrier and 1.65-fold, nonbarrier) in MFI in TZM-bl target cells compared to the nontarget HeLa cells (Figure 3B and Supporting Information File 1, Figure S4). Further, we observed similar trends for the A-1 aptamer, where Tat had no effect on BBB penetration (Figure 3C, Figure 3D, and Supporting Information File 1, Figure S4). Interestingly, in this group, percentage uptake was lower across all groups compared to the LNP A-1 T7 group (Figure 3C and Figure 3D). This may be due to differences in hCMEC/D3 barrier formation or LNP counting error using NTA.

Collectively, these data suggest that the addition of Tat to LNPs has no effects on BBB transit when compared to the T7 peptide. We further found that A-1 aptamer incorporation into the LNP formulation does not appear to enhance specific targeting of gp160-expressing cells either through the hCMEC/D3 barrier or through direct addition, suggesting that it may not be an ideal candidate moving forward.

### LNPs do not stimulate an immune response

**Figure 4:**
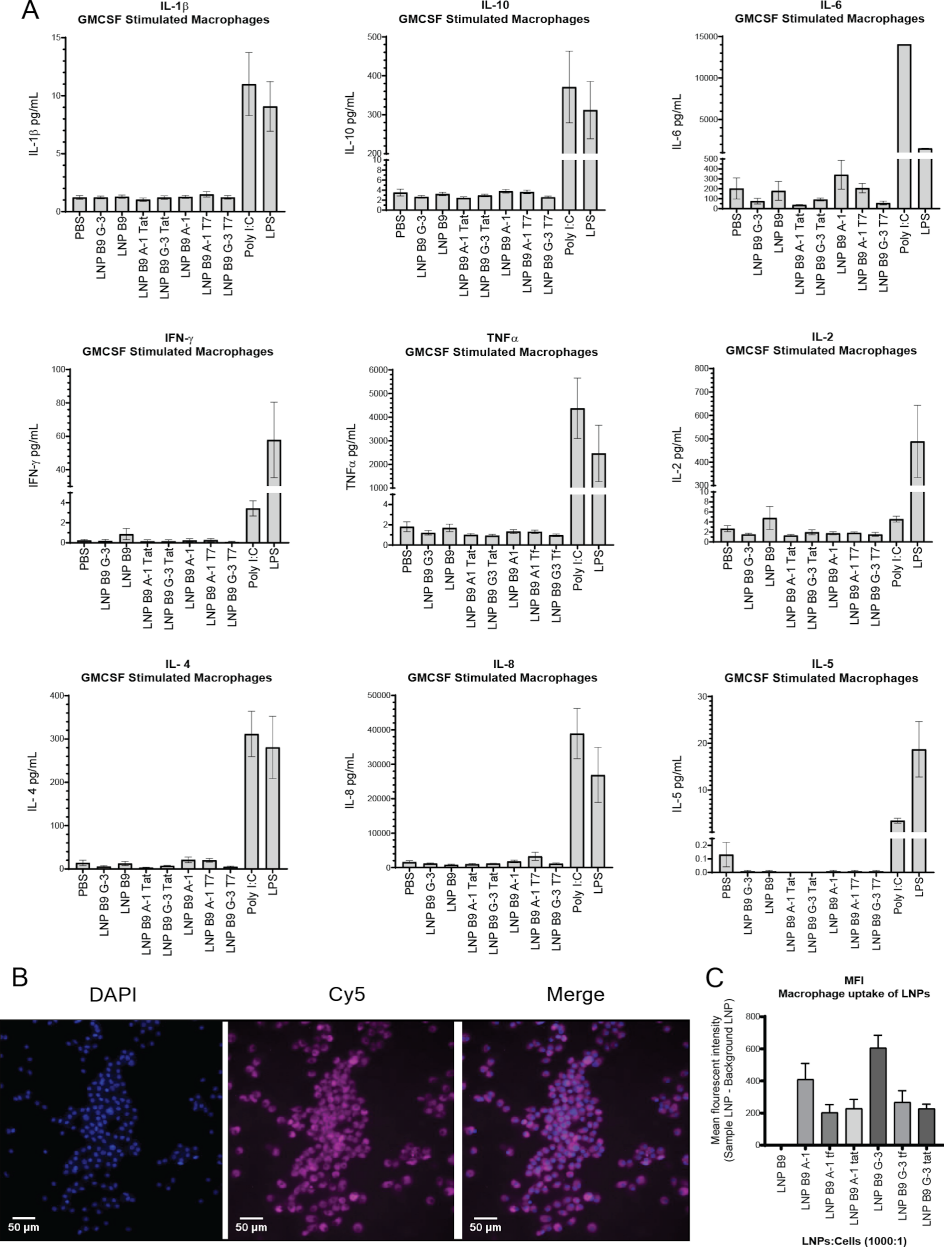
LNPs do not stimulate secretion of proinflammatory cytokines. A) GMCSF-primed MDMs were treated with LNPs at a ratio of LNPs/cells 1000:1 or with poly I:C or LPS for 24 h. Thereafter, supernatants were harvested, clarified, and processed for cytokine detection by Luminex. Analytes included IL-1β, IL-10, IL-6, IFN-γ, TNFα, IL-2, IL-4, IL-8, and IL-5. Histograms are representative of three biological experiments, each containing duplicate technical replicates. B) Representative fluorescent images (4′,6-diamidino-2-phenylindole (DAPI), Cy5, and merged) of macrophages and LNP G-3 after 24 h. All macrophages were 100% positive for LNP uptake independent of aptamer and peptide composition. C) However, MFI analysis using QuPath v0.2.2 suggests that the LNP G-3 had the highest uptake compared to the other LNP formulations in type-1 MDMs.

In order to further characterize LNPs, we decided to evaluate the immunogenic profile. We stimulated monocytes obtained from whole blood for 6 days with 10 ng/mL granulocyte-macrophage colony-stimulating factor (GM-CSF). This programs the monocytes to form macrophages that are primed to respond in a type-1 manner. After 24 h of stimulation with either the LNPs or positive controls for an RNA/DNA response (poly I:C) or a bacterial response (LPS), we found that the LNPs did not increase secretion of any of the cytokines tested (IL-1β, IL-10, IL-6, IFN-γ, TNFα, IL-2, IL-4, IL-8, and IL-5) above basal (phosphate-buffered saline, PBS) conditions (Figure 4A). Additionally, we confirmed LNP uptake by the monocyte-derived macrophages (MDMs) using fluorescent microscopy (Figure 4B). We found that all LNPs containing the Cy5 oligonucleotide were observable under the microscope (Figure 4B) and that all macrophages were 100% positive for Cy5. Additionally, using QuPath analysis software, we determined the Cy5 MFI for each image. Interestingly, we found that the LNP A-1 and the LNP G-3 had higher MFI values in all the donors assessed compared to the Tat and T7 counterparts (Figure 4C). Further, we found that the LNP G-3 exhibited the highest uptake in all the donors assessed (Figure 4C). These observations suggest that the candidate LNPs are relatively immunologically inert and may prove to be well-tolerated in vivo.

### Aptamer and peptide LNPs have modest effects on cell viability in a cell-specific manner

**Figure 5:**
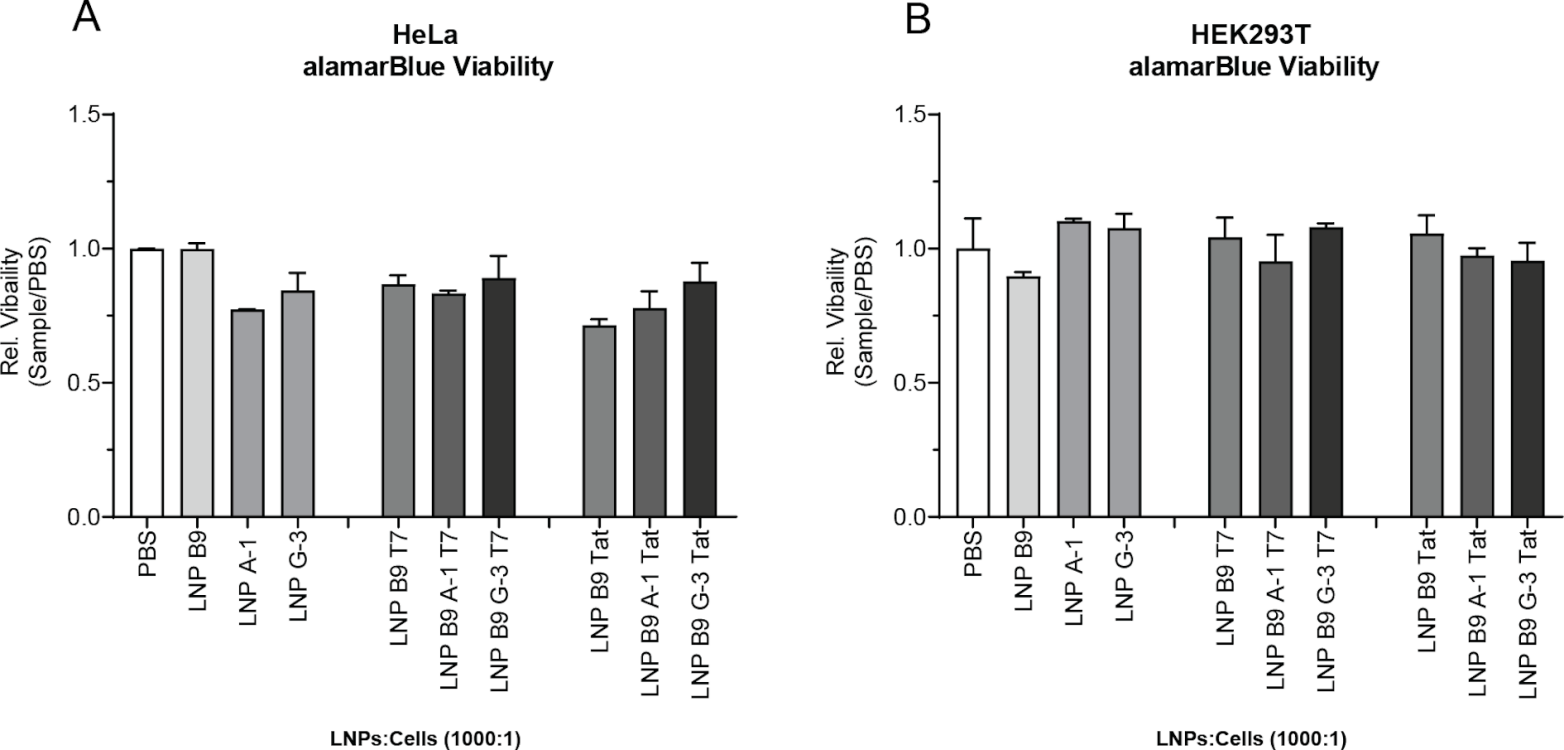
LNPs modestly affect cell viability in a cell-specific manner. HeLa (A) or HEK293T cells (B) were treated overnight with the LNPs at a ratio of 1000:1. Next, the alamarBlue viability assay was performed and viability as fraction of control (using PBS) was determined. Histograms are representative of the mean ± SEM. Data representative of two independent experiments performed in quadruplicates.

We next assessed whether LNPs could affect cell viability in HeLa and HEK293Ts cells. Cells were treated with the LNPs for 24 h prior to performing the alamarBlue viability assay. In HeLa cells, we found that the LNP B9 alone had no effect on cell viability compared to the PBS control (Figure 5A). Interestingly, we observed that cell viability was reduced by ≈20% in HeLa cells treated with LNPs containing either A-1 or G-3 aptamer or LNPs with the Tat or T7 peptide alone (Figure 5A). However, LNPs containing both the aptamer and a peptide (Tat or T7) did not further affect cell viability (Figure 5A). This suggests that the aptamer and the peptides may contribute towards the loss of cell viability observed in this cell type. Conversely, we observed no loss of cell viability in HEK293T cells treated with LNPs containing either A-1 or G-3 aptamer, Tat or T7 alone, or the combination of aptamers and peptides (Figure 5B). As observed in the HeLa cell line, the LNP formulation alone had no effect on cell viability (Figure 5B). These data suggest that there may be some cell-specific sensitivity toward the LNPs formulations and that further studies are required to determine the optimal concentrations of aptamers and peptides within the LNPs or to optimize the ratio of LNPs to cells in order to reduce toxicity in any cell line tested.

## Discussion

LNPs represent an increasingly popular modality for cargo delivery. The vast improvements in lipid design and architecture have resulted in several successful LNP-driven vaccines and therapeutics, including two RNA-based severe acute respiratory syndrome coronavirus 2 (SARS-CoV-2) vaccines [46], as well as an siRNA–LNP for the treatment of a transthyretin amyloidosis [36]. However, further improvements in toxicity profiles, cargo delivery, and cell or organ specificity are needed to expand the use of LNPs for gene and drug delivery.

LNPs and aptamers have previously been used with great success to increase cell specificity. In 2015, Liang et al. reported on a novel aptamer–LNP targeting osteoblasts. The authors conjugated aptamers to a 1,2-distearoyl-*sn*-glycero-3-phosphoethanolamine-*N*-[amino(polyethylene glycol)-2000] and observed that their LNP–DNA aptamer was able to deliver target siRNA into osteoblasts via macropinocytosis, increasing bone formation in vitro and in rodents [47]. In 2017, Kim et al. used a postinsertion method to incorporate an aptamer–maleimide–PEG into their LNPs to target epidermal growth factor receptor (EGFR)-positive cancer cells. The authors showed an increased delivery of siRNAs and fluorescent quantum dot nanocrystals both in vitro and in EGFR-positive tumor xenografts in mice [48]. In 2020, Chandra et al. used a maleimide–PEG in their LNP formulation to functionalize LNPs with an aptamer specific for the human epidermal growth factor 2 (HER2) receptor. Therein, functionalized LNPs increased siRNA delivery and subsequent sensitivity of the doxorubicin-resistant HER2-positive breast cancer cell lines by ≈2-fold over LNPs with no aptamers [49]. Taken together, the work reported by these authors, as well as by others, demonstrates the ability of aptamers to increase cellular specificity and uptake of LNPs into the target cells. In the present study, we observed that LNPs containing the G-3 aptamer targeting CCR5 resulted in a 40% increase in cellular uptake through the BBB and into target cells and that these cells had higher LNP uptake (measured by a higher MFI) than the non-antigen-expressing counterparts, while the gp160 aptamer (A-1) had no apparent effect on target cell uptake. One could speculate that this may be the result of the nature of the target proteins. CCR5, a cell surface receptor, is internalized upon ligand binding before recycling back to the cell surface or processed for degradation in the lysosome [34]. On the other hand, gp120 is a viral surface protein that is involved in viral entry through complexation with cluster of differentiation 4 (CD4) and CCR5 or C-X-C motif chemokine receptor 4 (CXCR4) host cell surface receptors [35]. As such, gp160 expression on the host cell surface receptor may not be as adept at facilitating cell entry via receptor-mediated endocytosis. Although in 2009, Zhou et al. observed by confocal microscopy that the A-1 aptamer entered gp160-positive cells and suggested that receptor-mediated endocytosis could be the mechanism of entry, such a notion was not definitively demonstrated as the mechanism of uptake [35]. In addition, observed differences between these aptamers could also be due to differences in target receptor expression in the cell types and/or differences in the affinity and specificity of these aptamers for the target receptors and/or differences in the mechanisms of uptake. Finally, the formulation procedure also likely influences the ability of the aptamers to act as productive ligands for the respective receptors, although more studies will be needed to fully delineate these effects.

One important aspect we set out to address was to identify proxies for successful LNP-mediated cargo delivery through the BBB and into the brain. As previously stated, effective transport systems for brain drug delivery are highly warranted. Herein, we find that the LNP platform can be applied as a vehicle to circumvent the BBB and effectively deliver oligonucleotide probes to antigen-expressing cell lines. For HIV-1 there is currently a need for more effective delivery platforms compatible with antiretroviral drugs. Specifically, a productive CNS delivery of such compounds is expected to reduce HIV-1-associated neurological disorders as well as to reduce HIV-1 replication at this sanctuary site [13,50–51].

We investigated the use of T7 and Tat peptides and evaluated the ability of these to aid delivery of LNP–aptamer species across the BBB. We found that LNPs with either T7 or the Tat peptide did not significantly increase cellular uptake through the BBB above the LNPs-containing aptamers alone. T7 appeared to have an effect on cellular uptake when the LNPs were directly added to the cells and a small effect when applied through the apical chamber of the hCMEC/D3 cell line, while Tat had no effect. It may be prudent to dose the amount of postinserted Tat or T7 peptide used in these formulations. For example, in 2007, Duchardt et al. used 2–40 µM Tat peptide as a cell-penetrating peptide to facilitate siRNA entry. In particular, the authors observed that clathrin-dependent endocytosis increased with increasing concentrations of Tat peptide, suggesting that a high concentration may be needed to elicit an effective endocytosis mechanism [18]. In 2011, Qin et al. conjugated Tat to PEG 2000 and found that in their liposomal formulations, those containing 10% PEG 2000–Tat had the most efficient uptake in a BBB model [9]. Several studies have used transferrin-conjugated PEG analogs. In 2011, in a series of papers, Pang et al. observed that liposomes comprised of 5–10% PEG–transferrin increased brain delivery by 2.8-fold compared to liposomes without transferrin in a BBB model and in vivo [15] and further when loaded with the chemotherapeutic agent doxorubicin. They observed increased delivery of this compound and subsequently a significant tumor regression in mouse xenografts [22]. In 1997 and 2002, Kircheis et al. developed a polyethyleneimine (PEI)-conjugated transferrin molecule at a ratio of PEI/transferrin 21.4 nmol:270 nmol and observed that transferrin shielded the PEI, decreasing toxicity and increasing target cell uptake through binding to the transferrin receptor both in vitro and in vivo [52–53].

In the work presented here, we immobilized Tat or transferrin onto the LNP formulations using a postinsertional technique. It could be that it would be more prudent to make the LNP formulation with the addition of Tat and T7 peptide during the initial synthesis. Based on the literature [22], we assume that endocytosis plays a role in the uptake of the decorated LNPs developed herein. Further, it may be important to increase the amount of postinserted Tat and T7 used in future experiments, considering the concentration we used was relatively low (≈0.1% postaddition). Another approach is to use a next generation of short peptides that also bind to the transferrin receptor at noncompeting regions to endogenous transferrin in vivo [54]. These molecules are known as cysteine-dense peptides (CDPs) and have been shown to bind to the transferrin receptor in the picomolar range to facilitate BBB crossing in mouse models [54]. These short peptides may be advantageous to use when approaching an in vivo strategy, especially considering that the concentration of the peptide needed in the formulation may be lower compared to the T7 peptide used in this study; however, its safety profile must still be fully evaluated.

Nevertheless, our LNPs, particularly the ones containing the G-3 aptamer alone, resulted in BBB transport ranging from 50–65% in nontarget cell lines to 80–100% uptake in target cell lines, suggesting that this is a viable approach to improve uptake. Importantly, the hCMEC/D3 model represents a simplified representation of the BBB, which does not account for the full complexities of the BBB in vivo [43,55]. One could perform more complex in vitro assays that include a multicellular reconstruction of the BBB to also include astrocytes and microglial cells [56–57]. Cellular studies could also reveal in detail the uptake mechanism of our LNPs. However, it may be more effective to perform further studies in nonprimate animal models to determine the efficacy of these LNPs in passing through the BBB. Assessing and quantitating the percentage of LNP B9 to traverse the BBB is a critical step to determine the use as an effective LNP able to deliver small molecules or oligonucleotides into the brain. One important caveat to note is that the aptamers are species-specific, and thus the use of a xenograft model with human cells in a nonprimate animal model is needed to determine the specificity of the LNP–aptamer tested.

Furthermore, while the LNP B9 alone had no effect on cellular viability, it appeared that the LNPs containing either the A-1 or G-3 aptamers, or the peptides, reduced cellular viability in HeLa cells by 20%, suggesting that there may be some toxicity when delivered to cells. However, these effects were not observed in HEK293T cells. It could be that the HeLa cell line is more sensitive than the HEK293T cell line. Nevertheless, the data suggest that further testing is required to determine the safety profile of these LNP aptamer and/or peptide formulations. One way we could reduce the toxicity profile is to chemically modify the RNA aptamers [33,58], or by reducing the aptamer concentration per LNP to thereby alleviate some of the observed cellular toxicity. It could be that the RNA aptamer itself could contribute towards cell death, possibly through stimulating the retinoic acid-inducible gene 1 (RIG-1) pathway, and it may thus be prudent to assess type-I interferons (IFN-α and IFN-β) in the future [32,59]. Importantly, the LNP B9 formulation alone had no effect on cell viability, suggesting that the ratio of cationic and ionizable lipids is optimal and does not present acute toxicity issues. However, more work is needed to assess the toxicity in vivo and, in particular, to evaluate the effect on the liver [60]. Importantly, the LNPs reported herein did not appear to stimulate an immune response in primary human monocyte-derived macrophages. Further, the addition of the aptamers and/or the peptides in the LNP formulations had no effect on immune stimulation, suggesting that these LNPs and the modifications may be well-tolerated in vivo. Importantly, both IL-6 and IFN-γ cytokines were not stimulated after exposure to the LNPs, suggesting that this LNP formulation may not induce cytokine release syndrome in vivo [60–61].

## Conclusion

Taken together, we have shown that the LNP B9 formulation is safe, can traverse the BBB, and is readily taken up in multiple cell types. In the future, it will be interesting to explore whether increased uptake may also lead to increased delivery of target molecules, such as siRNA, mRNA, or small molecules. Further, having LNPs that are specific for HIV-1-infected cells or HIV-1 target cells, may help to facilitate HIV-1 drug treatment to regions of poor drug accessibility, such as the brain. More effective delivery of antiretroviral drugs may help to reduce HIV-1-associated neurological disorders that are present in HIV-1-positive individuals as well as to reduce populations of HIV-1-positive cells that are poorly accessible through current systemic drug treatment strategies.

## Experimental

### Materials

(6*Z*,9*Z*,28*Z*,31*Z*)-Heptatriaconta-6,9,28,31-tetraen-19-yl 4-(dimethylamino)butanoate (DLin-MC3-DMA, >98%) was purchased from D&C Chemicals (China), DSPC and cholesterol were purchased from Echelon Biosciences, Inc. (USA), and DMG-PEG 2000 was purchased from Avanti Polar Lipids, Inc. (USA). Ethanol (BioUltra, ≥99.8%), citric acid monohydrate, sodium chloride, Na_2_HPO_4_, and KH_2_PO_4_ were purchased from Sigma-Aldrich (Germany).

### RNA and DNA oligonucleotides

The RNA aptamers and Cy5 DNA oligonucleotides were synthesized and purified using ion-paired and ion-exchange HPLC at the RNA/DNA synthesis core at City of Hope (Duarte, CA). The RNA aptamers, A-1 [35] and G-3 [34], were developed by Dr. Jiehua Zhou at City of Hope (Duarte, CA), and include the addition of a 3 carbon linker (*XXXXXX*), a sticky-bridge motif, and a 3’ amino linker C6, 3aminoC6, at the 3’ end. Annealing of the Cy5 DNA oligonucleotide to the sticky-bridge motif of the RNA aptamers was confirmed using an electromobility shift assay (EMSA) using an 8% tris/borate/ethylenediaminetetraacetic acid (TBE)-buffered gel (Novex™ Thermo Fisher Scientific, MA), under native conditions (Supporting Information File 1, Figure S1A).

#### RNA aptamers

- A-1 or GP160: 5’- GGG AGG A**C**G A**U**G **C**GG AA**U U**GA GGG A**CC** A**C**G **C**G**C U**G**C UU**G **UU**G **U**GA **U**AA G**C**A G**UU U**G**U** CG**U** GA**U** GG**C** AGA **C**GA **CUC** G**CC C**GA *XXXXXX G***U***A***C***A***U UCU***AGA***U***AG***CC** /3aminoC6 -3’
- G-3 or CCR5: 5’- GGG AGG A**C**G A**U**G **C**GG G**CC UUC** G**UU U**G**U UUC** G**UC C**A**C** AGA **C**GA **CUC** G**CC C**GA *XXXXXX ***U***GA* **U***AG A***UU** *GA***U** *AGA* /3aminoC6 -3’

Bold = 2’-flouronated base; italics and underlined = 2’-*O*-methyl base

#### Complementary DNA

- A-1: (Cy5/AGG CTA TCT AGA ATG TAC)
- G-3: (Cy5/TCT ATC AAT CTA TCA)

### Peptide synthesis, purification, and characterization

Peptide assembly was carried out by solid-phase peptide synthesis in standard solid-phase extraction filtration columns. Initially, fluorenylmethyloxycarbonyl (Fmoc) group removal from the Rink linker was achieved by applying 20% piperidine in DMF (2 × 30 min). Preactivation of Fmoc amino acid (4 equiv) prior each coupling was performed with 1-[bis(dimethylamino)methylene]-1*H*-1,2,3-triazolo[4,5-*b*]pyridinium 3-oxide hexafluorophosphate (HATU, 4 equiv) and *N*,*N*-diisopropylethylamine (DIPEA, 6 equiv) in DMF. Then, the activated mixture was added to the resin swollen in DMF, and manual stirring was applied approximately every 15 min over a total reaction time of 2 h. The first amino acid was installed via double coupling. Fmoc deprotection was achieved via 20% piperidine in DMF (1 × 2 min and 1 × 18 min) to prepare the resin for the next coupling step. The resin was washed three times with each solvent in the given order DMF, DCM, and DMF after every reaction step.

#### Peptide sequences

- T7: H-HAIYPRH-NH_2_
- Modified T7: dipalmitoyl-Dap-HAIYPRH-NH_2_
- Tat: H-YGRKKRRQRRR-NH_2_
- Modified Tat: dipalmitoyl-Dap-YGRKKRRQRRR-NH_2_

### Peptide conjugation with a lipid reagent

The peptides were N-terminally modified on solid support by coupling of Fmoc-Dap(Fmoc)-OH, followed by the coupling of palmitic acid to afford the complete peptide–lipid conjugates. Coupling of Fmoc-Dap(Fmoc)-OH and Fmoc deprotection were carried out as described above. To ensure the complete lipidation of the two free amines of Dap, 8 equiv of palmitic acid, 8 equiv of HATU, and 12 equiv of DIPEA in DMF were used. Cleavage of the peptide–lipid conjugates from the solid support and removal of the side-chain protecting groups was achieved by using trifluoroacetic acid (TFA)/phenol/water/triisopropylsilane (TIPS) 88:5:5:2 (3 × 60 min). After cleavage, the remaining resin was extracted with DCM (2 × 10 min). All DCM extracts and TFA cleavages were combined, and the resulting mixture was reduced under nitrogen flow. The received solid product was dissolved in DCM and subsequently reduced under nitrogen flow. This procedure was repeated two more times, followed by a lyophilization step to receive the crude peptide. The crude T7–lipid conjugate was purified by normal-phase chromatography utilizing gradient elution (2–50% MeOH in DCM). The desired modified T7 peptide was characterized via MALDI-TOF spectrometry (Bruker, MA, Supporting Information File 1, Figure S1B) and isolated as a colorless powder (9 mg, 6 μmol, 6% yield). MS (*m*/*z*): [M + H]^+^ calcd, 1455.00; found, 1455.20. The crude Tat–lipid conjugate was precipitated from DMF as a white power and used without further purification. The modified Tat peptide was characterized via MALDI–TOF spectrometry (Supporting Information File 1, Figure S1C, 31 mg, 14 μmol, 15% yield). MS (*m*/*z*): [M + H]^+^ calcd, 2121.48; found, 2121.17.

### Lipid nanoparticle synthesis

The formulation protocol was largely adapted from Jayaraman et al. (2012) [4]. Freshly prepared lipid stocks (in chloroform) were mixed to obtain the desired mole fractions (DLin-MC3-DMA/DSPC/Cholesterol/DMG-PEG 2000 0.4:0.1:0.4:0.1), and the lipid mixture was concentrated under vacuum. The lipid film was dissolved in ethanol (20.3 mg/mL) and added dropwise to stirring 50 mM citrate buffer at pH 4.0 and preheated to 35 °C to get a final lipid concentration of 6.1 mg/mL. The lipid solution was stirred for an additional 20 min at 35 °C, after which the lipid solution was allowed to slowly reach rt, transferred to a 1 mL Hamilton syringe, and extruded 10 times at rt through two 100 nm Nucleopore membrane filters (Whatman) using Avanti Mini Extruder (Avanti Polar Lipids, Inc., USA).

Complementary oligonucleotides GP160:A-1 (1.4 nmol, 30 μL 1× PBS pH 7.4) and CCR5:G-3 (1.4 nmol, 30 μL 1× PBS pH 7.4) underwent annealing (85 °C for 10 min, 25 °C for 20 min, 4 °C for 20 min). GP160:A-1 (30 μL) and CCR5:G-3 (30 μL) were each added to a stirring LNP suspension (6.1 mg/mL, 165 μL) preheated to 35 °C, and LNP–DNA lipoplex suspensions were further diluted with 50 mM citrate buffer at pH 4.0 and 30% EtOH (120 μL), to get a final lipid concentration of 3.2 mg/mL and a DNA/lipid ratio of roughly 0.05 w/w. The LNP–DNA lipoplexes were allowed to form over 30 min at 35 °C (no stirring). Buffer exchange was performed using 3K Amicon Ultra-0.5 Centrifugal Filter Unit (Merck Millipore, USA), providing the final LNP–DNA lipoplexes in 1× PBS pH 7.4 (3 mg/mL final lipid concentration). Postinsertion of peptides was carried out by diluting the peptides to a final concentration of 1.7 µg/mL Tat lipid and 3.0 µg/mL T7 lipid in 1× PBS pH 7.4. Thereafter, diluted lipopeptides (18 µL T7, 31.8 µL Tat) were added to the LNPs (90 µL). Samples were incubated on a thermomixer for 30 minutes (25 °C at 250 rpm) for postinsertion addition. Thereafter, samples were stored at 4 °C until further use.

### DLS and ZP

Particle size, polydispersity, and ZP were analyzed by DLS instrument model Zetasizer Nano ZS (Malvern Instruments, UK), having He–Ne 633 nm laser at an angle of detection of 90°, with an incubation time of 60 s. Samples were diluted 50-fold in Milli-Q water and placed into the disposable plastic cuvettes for measurement performed in triplicates (*n* ≥ 3) to obtain a mean value.

### NTA

Concentration and size of LNPs with and without peptides were additionally confirmed using the NanoSight NS300 device (Malvern Panalytical, UK) with the NTA software (Version 3.44, Malvern Panalytical, UK). Samples were run at a 1:1000 dilution, with three technical replicates per sample. A blue 488 nm laser was used to detect the LNPs, with a slide shutter level set to 1232 and the slider gain set to 219, and the syringe pump speed set to 30 using a flow-cell top plate module.

### Cell lines and maintenance

HeLa and HEK293T cells were purchased from American Type Culture Collection (ATCC, VA). TZM-bls were acquired through the NIH AIDS reagent program and were engineered to express high levels of the HIV-1 coreceptor CCR5 [44]. HEK293T-gp160 cells were a kind gift from Dr. Bing Chen (Harvard, MA), and stably expressed the 92UG037.8 strain of the viral envelope protein, Env [45]. The human brain endothelial cell line hCMEC/D3 was purchased from Millipore Sigma (MA). HeLa, TZM-bls, HEK293T, and HEK293T-gp160 cell lines were all cultured in Dulbecco’s Modified Eagle’s Medium (DMEM, Corning™, NY) in 10% fetal bovine serum (FBS, GeminiBio, CA). The hCMEC/D3 cell line was maintained in EndoGRO-MV complete culture medium (Millipore Sigma, MA) on collagen (collagen type 1, rat tail, Millipore Sigma, MA)-coated flasks. hCMEC/D3 cells were cultured to a maximum of 10 passages to ensure proper tight junction formation. All cells were maintained in a water jacket incubator at 37 °C. All cell lines were routinely tested and found negative for mycoplasma.

### Inflammation assay

Blood from consented and deidentified donors was used in this study under an approved IRB 19582 (City of Hope, Duarte, CA). To obtain monocytes, we followed the methodology by Menck et al. of 2014 [62]. Briefly, blood was initially processed using a Histopaque^®^-1077 (Millipore Sigma, MA) density separation to collect the buffy coat. Thereafter, the buffy coat was subject to a Percoll^®^ (Cytiva, MA) density separation to enrich for the monocyte population in the buffy coat. Monocytes were counted and stored in Cyrostor-C5 (BioLife Solutions, WA) at −80 °C until further use. Monocytes were plated at a density of 1 × 10^5^ cells per 96-well plate and stimulated for 6 days with 10 ng/mL GM-CSF (Gibco™, Thermo Fisher Scientific, MA) in Roswell Park Memorial Institute (RPMI) medium (Corning™, NY) supplemented with 5% FBS, 1% AB normal human serum (Millipore Sigma, MA), and 1% penicillin/streptomycin (Millipore Sigma, MA). The medium was replaced every 3 days. After 6 days, the medium was replaced without GM-CSF, and LNPs (ratio 1000:1), poly I:C (25 µg/mL, Millipore Sigma, MA), or LPS (1 µg/mL, Millipore Sigma, MA) was added to the macrophages. 24 h later, the supernatant was collected and centrifuged at 300*g* for 5 minutes to remove cellular debris. Harvested supernatant was stored at −80 °C until processed for cytokine expression using a 10-Plex Human Cytokine Panel (LHC6004M, Thermo Fisher Scientific, MA). The Luminex assay was processed on a Luminex^®^ 200 machine (Luminexcorp, TX) by the Analytical Pharmacology Core (City of Hope, Duarte, CA).

### Transwell assay

The transwell assay was adapted from Weksler et al. (2005) [55]. Briefly, hCMEC/D3 were cultured on presoaked 0.4 µM transwell filters (Greiner Bio-One Thincert^™^ CellCoat^™^, Austria) at a density of 5 × 10^4^ cells/cm^2^ in a 24-well culture dish. After 6 h, the medium was removed from the apical chamber and replaced with 200 µL fresh EnoGRO-MV complete culture medium (Millipore Sigma, MA). The basolateral chamber was filled with 600 µL medium. The next day, the medium was changed to a low-supplement endothelial cell growth medium-2 (EGM-2) basal medium (Lonza Walkerville, MD) supplemented with 2.5% FBS, 0.55 µM hydrocortisone (Stemcell Technologies, Canada), 1% penicillin/streptomycin (Millipore Sigma, MA), and 10 mM 4-(2-hydroxyethyl)-1-piperazineethanesulfonic acid (HEPES, Gibco™, Thermo Fisher Scientific, MA). The culture was maintained and the medium replaced every 2nd day until a TEER of ≈30 Ω⋅cm^2^ was reached. TEER was measured using an EVOM2 with a chopstick electrode (World Precision Instruments, FL). Resistivity was calculated using the formula given in Equation 1.

[1] $$\text{resistivity }(\Omega \cdot {\text{cm}}^{2})=(\text{sample }\Omega -\text{control }\Omega )\times \text{surface area of insert}$$

Once the integrity of the barrier was assessed, the apical transwell chambers were transferred to new 24-well culture dishes with 50,000 cells per well of HeLa, TZM-bl, HEK293T, or HEK293T-gp160 cells that had been plated 24 h previously. An LNPs/cells ratio of 1000:1 was added to each well. 24 h later, the apical layer was removed, and the basolateral cells were washed, trypsinized, and resuspended in 1× PBS. Detection of Cy5 was measured by flow cytometry on a BD Accuri™ C6 device (Becton, Dickinson and Company, NJ), and the data was analyzed using FlowJo™ Version 10.7.1 (Becton, Dickinson and Company, NJ). Cells were first gated on forward scatter-area (FSC-A) vs side scatter-area (SSC-A), followed by side scatter-height (SSC-H) vs SSC-A to gate on single cells, before designating negative and positive population gates using a histogram.

### Viability assay

The alamarBlue assay was performed according to the manufacturer’s instructions (Thermo Fisher Scientific, MA). Briefly, 10,000 HeLa cells and 40,000 HEK293T cells were seeded in a 96-well plate. The next day, LNPs at a ratio of 1000:1 were added to the cells. 24 h later, 0.1 volume of 10× alamarBlue was added and the cells incubated for 1 h at 37 °C. Fluorescence was measured on a GloMax^®^ Explorer multimode microplate reader (Promega, WI). Background measurements from a medium-only control were subtracted from all the measurements before calibrating to the PBS control.

### Light microscopy

To assess tight junction formation, we adapted the protocol from Vu et al. of 2009 [43]. Briefly, the apical chamber was washed with 1× PBS and fixed with ice-cold 4% paraformaldehyde for 15 minutes at 4 °C before washing two times with ice-cold PBS. The chambers were blocked with 1% bovine serum albumin (BSA)–PBS for 60 min at 4 °C and subsequently incubated overnight at 4 °C with Claudin 5–Alexa Fluor 488 (catalog number 35-258-8, Thermo Fisher Scientific, MA) at 5 µg/mL in 1% BSA–PBS. Thereafter, cells were washed three times with ice-cold PBS. The membrane was subsequently cut out of the insert with a scalpel blade and, using tweezers, placed on a slide and air-dried. Once dried, a small drop of Diamond Anti-Fade Mountant with DAPI (Invitrogen™, Thermo Fisher Scientific, MA) was added and a coverslip placed over the membrane. Slides were cured overnight at 4 °C before being visualized using a Zeiss Axio Vert A.1 light microscope with a Zeiss AxioCam 503 color camera (Carl Zeiss Microscopy GmbH, Germany). Images were processed using ZEN blue software (Version 2.3, Carl Zeiss Microscopy GmbH, Germany) and merged using ImageJ Version 1.53a (Wayne Rasband, NIH, USA).

To assess uptake of the LNPs in primary macrophages, samples were washed once with PBS and fixed with 4% ice-cold paraformaldehyde (in PBS) for 15 minutes at 4 °C. The formaldehyde was removed and the cells washed twice with ice-cold PBS. Thereafter, PBS containing DAPI (10 ng/mL) was added and the cells visualized using a Zeiss Observer II light microscope with a Zeiss AxioCam 506 Mono camera (Carl Zeiss Microscopy GmbH, Germany). Images were acquired using the ZEN blue software (Version 2.3, Carl Zeiss Microscopy GmbH, Germany). Images were processed using ImageJ Version 1.53a (Wayne Rasband, NIH, USA). To analyze the mean fluorescent intensity, we used QuPath v0.2.2 [63] (The University of Edinburgh, UK). We analyzed two different fields of view per treatment group for each donor (*n* = 3). For the analysis, we used the positive cell detection software with the following parameters: detection channel set to DAPI with a requested pixel size of 0.45 µm. Nucleus parameters were set to a background radius of 8 µm, a media filter radius of 1 µm, a sigma value of 3 µm, a minimum area of 10 µm^2^, and a maximum area of 400 µm^2^. Intensity parameters were set to a threshold of 150. Cell expansion was set to 5 µm. “Split by shape”, “Include cell nucleus”, “Smooth boundaries”, and “Make measurements” boxes were all checked. Intensity threshold parameters were set to a single threshold with the score compartment set to cytoplasm: Alexa Fluor 647 mean. Mean cytoplasm Alexa Flour 647 values were used and represented as mean ± SEM.

### Negative staining electron microscopy of LNPs

Specimens diluted at 1:1000 were absorbed onto glow-discharged, carbon-coated 200 mesh electron microscopy (EM) grids. Samples were prepared by conventional negative staining with 1% (w/v) uranyl acetate. EM images were collected with an FEI Tecnai 12 transmission electron microscope (Thermo Fisher Scientific, MA) equipped with a LaB6 filament and operated at an acceleration voltage of 120 kV. Images were recorded with a Gatan 2 × 2 k CCD camera (Gatan, Inc., CA) at a magnification of 21,000–26,000× and a defocus value of ≈1.5 μm. TEM images were analyzed using ImageJ version 1.53a (Wayne Rasband, NIH, USA). Briefly, the scale was set to the scale bar on the image, and the diameter for entire nanoparticles was measured in each image. At least 3 images per LNP formulation were used to determine the size distribution of the LNPs. Data are represented as a box and whisker plot, with min and max values representing the error bars.

### Statistical analysis

Experiments are representative of two or three biological repeats performed in technical duplicates, unless otherwise stated. Data are represented as histograms with mean ± SEM. Data was prepared and analyzed using GraphPad Prism for Windows Version 8.3 (GraphPad Software, CA).

## Supporting Information

File 1 EMSA and MALDI–TOF of oligonucleotides, TEM data for LNPs, hCMEC/D3 cell images, and FACS images.
