# Supplementary material for: Enhanced target cell specificity and uptake of lipid nanoparticles using RNA aptamers and peptides
Source: Beilstein J Org Chem. 2021 Apr 26;17:891–907. doi: 10.3762/bjoc.17.75 (PMC8093553; doi:10.3762/bjoc.17.75)
Supplement: File 1 — EMSA and MALDI–TOF of oligonucleotides, TEM data for LNPs, hCMEC/D3 cell images, and FACS images. [file Beilstein_J_Org_Chem-17-891-s001.pdf]

BEILSTEIN JOURNAL OF ORGANIC CHEMISTRY

Supporting Information

for

Enhanced target cell specificity and uptake of lipid nanoparticles using RNA aptamers and peptides

Roslyn M. Ray, Anders Højgaard Hansen, Maria Taskova, Bernhard Jandl, Jonas Hansen, Citra Soemardy, Kevin V. Morris and Kira Astakhova

Beilstein J. Org. Chem. **2021,** *17,* 891–907. doi:10.3762/bjoc.17.75

EMSA and MALDI–TOF of oligonucleotides, TEM data for LNPs, hCMEC/D3 cell images, and FACS images

License and Terms: This is a supporting information file under the terms of the Creative Commons Attribution License (https://creativecommons.org/licenses/by/4.0). Please note that the reuse, redistribution and reproduction in particular requires that the author(s) and source are credited and that individual graphics may be subject to special legal provisions.
The license is subject to the *Beilstein Journal of Organic Chemistry* terms and conditions: (https://www.beilstein-journals.org/bjoc/terms)

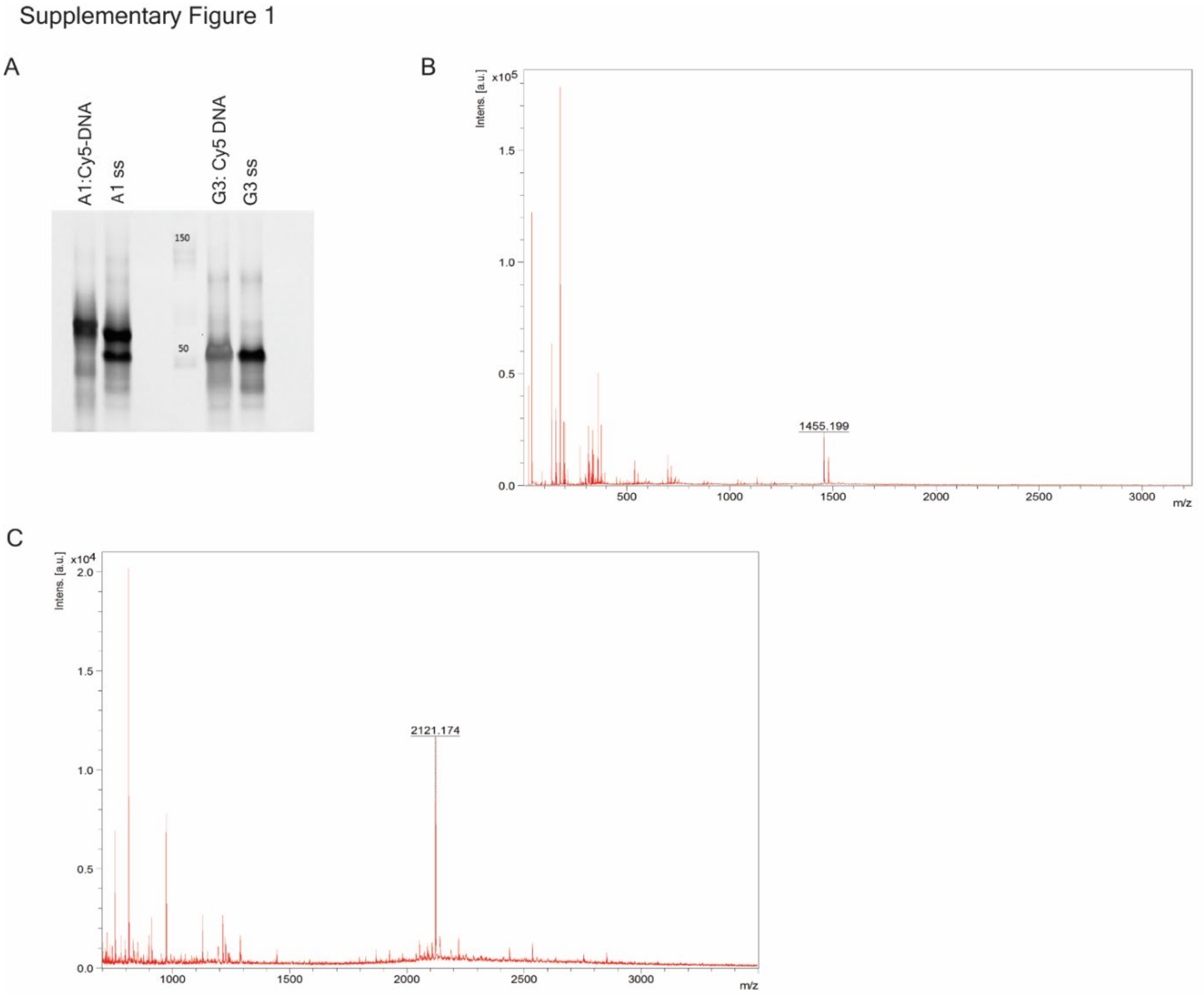

Figure S1: A) EMSA assay showing successful conjugation of the Cy5 DNA oligonucleotide to the RNA aptamers, A-1 and G-3. An 8% TBE gel (Novex™, Thermo Fisher Scientific, MA) was subject to electrophoresis for 30 minutes at 180 V under native conditions. The gel was soaked in a 2μg/mL EtBr solution for 10 minutes, washed three times with distilled water, and visualized using a Bio-Rad EZ Gel Doc system (Bio-Rad). B) and C) MALDI MS analysis of a lipid–peptide conjugate prepared in this study. B) The desired modified T7 peptide was characterized via MALDI-TOF spectrometry with $[M+H]^+$ calc. 1455.00, $[M+H]^+$ found 1455.20. C) The modified Tat peptide was characterized with $[M+H]^+$ calc. 2121.48, $[M+H]^+$ found. 2121.17.

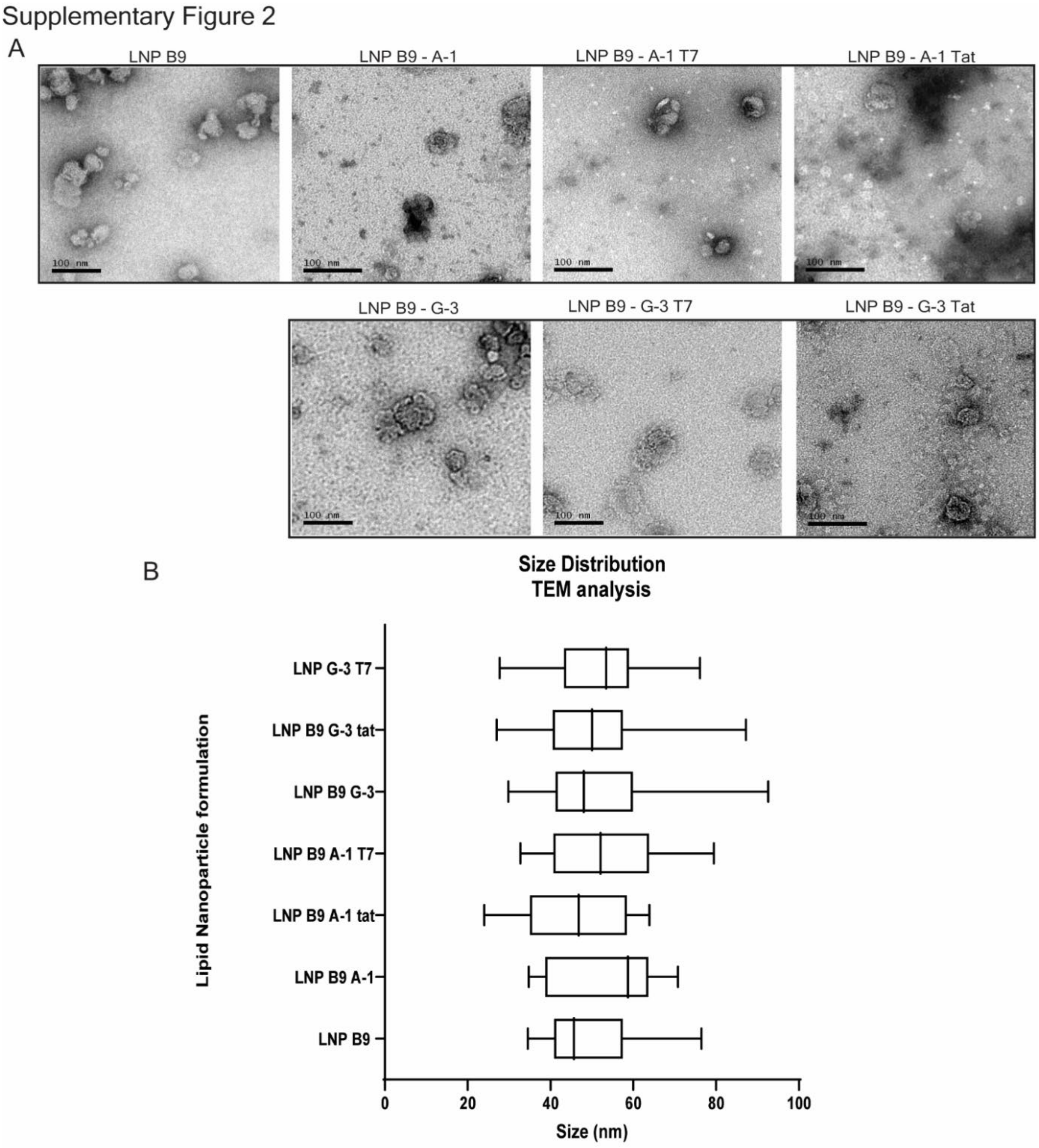

	LNP B9	LNP B9 A-1	LNP B9 A-1 tat	LNP B9 A-1 T7	LNP B9 G-3	LNP B9 G-3 tat	LNP G-3 T7
Mean	50.00	52.65	45.98	52.66	52.52	50.48	51.72
Std. Deviation	12.35	13.25	13.40	14.29	15.14	12.86	11.11
Std. Error of Mean	2.521	3.675	4.738	2.858	2.524	2.033	1.714

Figure S2: LNPs visualized using TEM. LNPs show consistent size and uniformity across different formulations. A) Representative images for each formulation and B) size distribution analysis. At least 3 images for each formulation were used to determine the

size distribution using ImageJ software (Version 1.53a, NIH). The sizes observed under TEM confirm the observations by DLS and NTA.

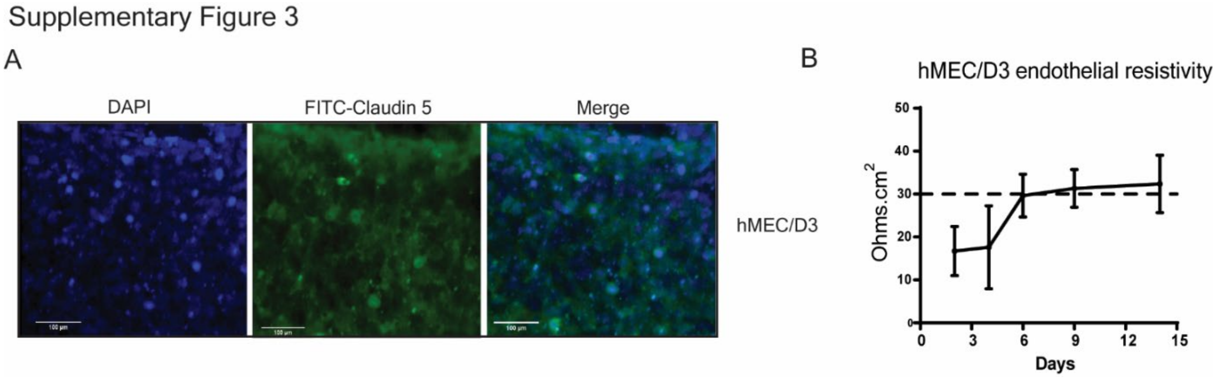

Figure S3: hCMEC/D3 cells were subject to immunofluorescence to determine the expression of the tight junction protein, Claudin-5, after culture in transwell apical chambers. Confirmation of Claudin-5 expression (A) suggests that a successful tight junction layer was formed in the in vitro culture. Further, resistivity was measured over time (B) to determine when the in vitro culture reached approximately 30 $\Omega \cdot cm^2$. This resistivity measurement also confirms tight junction formation in the hCMEC/D3 cell line in culture. Assays started when cultures had reached a resistivity of 30 $\Omega \cdot cm^2$ ranging between 6–12 days.

Supplementary Figure 4

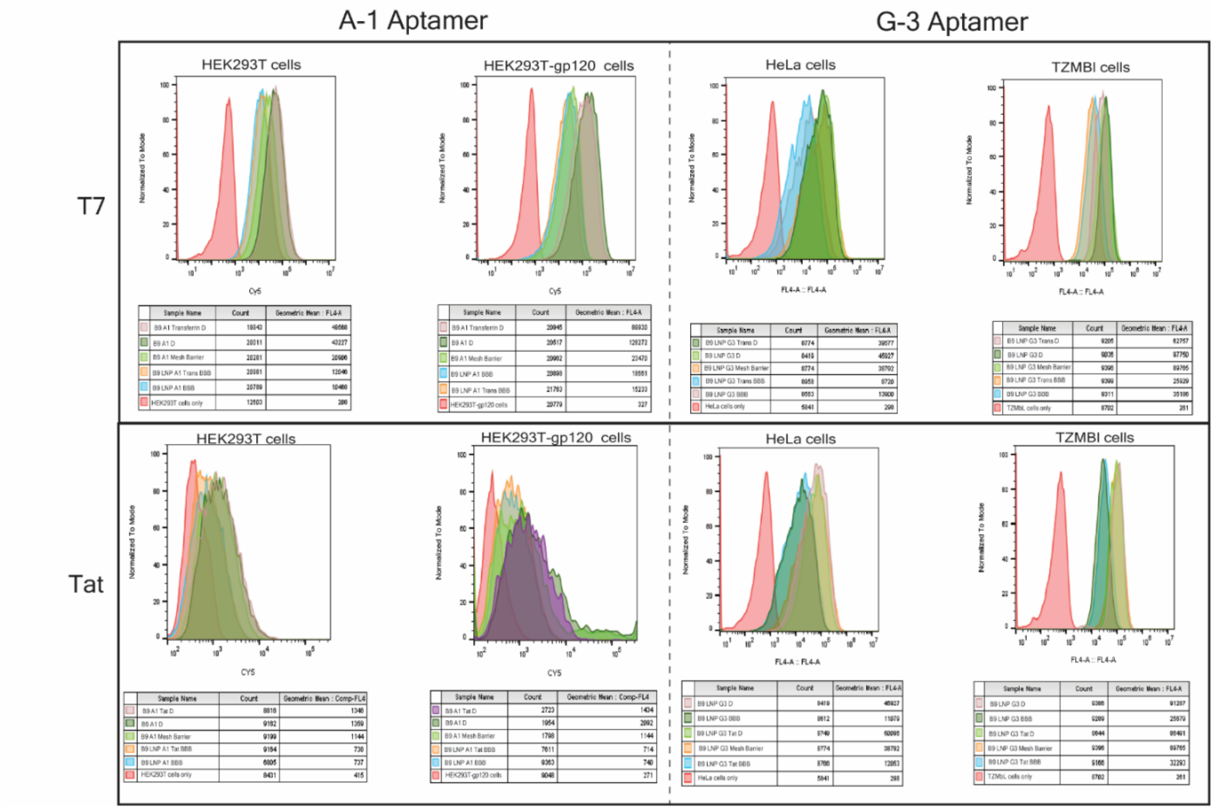

Figure S4: Histograms showing LNP uptake in the different cell lines and the corresponding MFI shifts. Top and bottom left are HEK293T cells and HEK293T-GP160-expressing cells exposed to LNP-A-1 with either T7 or Tat peptide postinserted. Top and bottom right are HeLa and TZMbl cells exposed to LNP-G-3 with either T7 or Tat peptide postinserted. Histograms show the increase in intensity and shift (to the right) in MFI intensity when the LNPs with aptamers are exposed to their target cells, compared to their nontarget cells.
